# Supplementary material for: Esterase mutation is a mechanism of resistance to antimalarial compounds
Source: Nat Commun. 2017 Jan 20;8:14240. doi: 10.1038/ncomms14240 (PMC5263872; doi:10.1038/ncomms14240)
Supplement: Supplementary Information — Supplementary Figures, Supplementary Tables and Supplementary Notes [file ncomms14240-s1.pdf]

Supplementary Table 1: Parasite Clones Resistant to Pepstatin Esters

Clone	Selection	Mutation in *pfpare* (PF3D7_0709700)	Amino Acid Change
3D7-R1	280 nM	T1070C	Leu357Pro
3D7-R2	280 nM	T968A	Leu323His
3D7-R3	1.2 μM	Δ1057	Ile353Leu, Ile354Ser, Thr355H, Trp356G, plus Δ358-368
3D7-R4	280 nM	C415T	Gln139Stop
HB3-R1	280 nM	G1068C	Trp356Cys
HB3-R2	280 nM	G1034T	Gly345Val
HB3-R3	1.2 μM	C910T	His304Tyr
HB3-R4	1.2 μM	T539A	Met180Lys

Resistant parasite clones were generated by continuously culturing *P. falciparum* strains 3D7 or HB3 with PBE. For clarity, the mutation numbers correspond to the coding strand of *pfpare*. EC_{50} values against PBE were > 10 μM for all clones.

Supplementary Table 2: *In Vitro* Activities of Pepstatin Analogs Against PfPARE Active Site Mutant Parasites

Compound	Potency* (μM) Strain: Wt**	Potency* (μM) Strain: S179T**
Pepstatin (P)	none at 40	N/D
Pepstatin methyl ester (PME)	5.19±0.24	none at 40
Pepstatin ethyl ester (PEE)	0.700±0.007	60% inhibition at 10
Pepstatin 3-methyl-1-butyl ester (PMBE)	0.076.2±0.001	none at 40
Pepstatin n-butyl ester (PBE) prep 2	0.107±0.008	none 10
Pepstatin n-hexyl ester (PHE) prep 2	0.024±0.004	40% inhibition at 1

* Potency - where appropriate, potency is presented as EC_{50}'s and measured against 3D7 asexual parasites using a flow cytometry based assay. For inactive compounds, % inhibition at highest concentration is presented.
** Wt is a clonal parasite line with *pfpare* endogenously expressed and containing a C-terminal GFP. S179T is a clonal parasite line with *pfpare* endogenously expressed, containing a C-terminal GFP and a S179T mutation.
± indicates standard errors. N=1-4.

Supplementary Table 3: Filter parameters for single nucleotide polymorphisms and insertions/deletions applied during full-genome sequencing.

SNP Filters

**Filter Name**	**Filter Value**
ReadPosRankSum	> 8.0
	< -8.0
QUAL	< 500
QD	< 2
MQRankSum	< -12.5
DP	< 5

INDEL Filters

**Filter Name**	**Filter Value**
ReadPosRankSum	< -20
QUAL	< 500
QD	< 2
DP	< 5

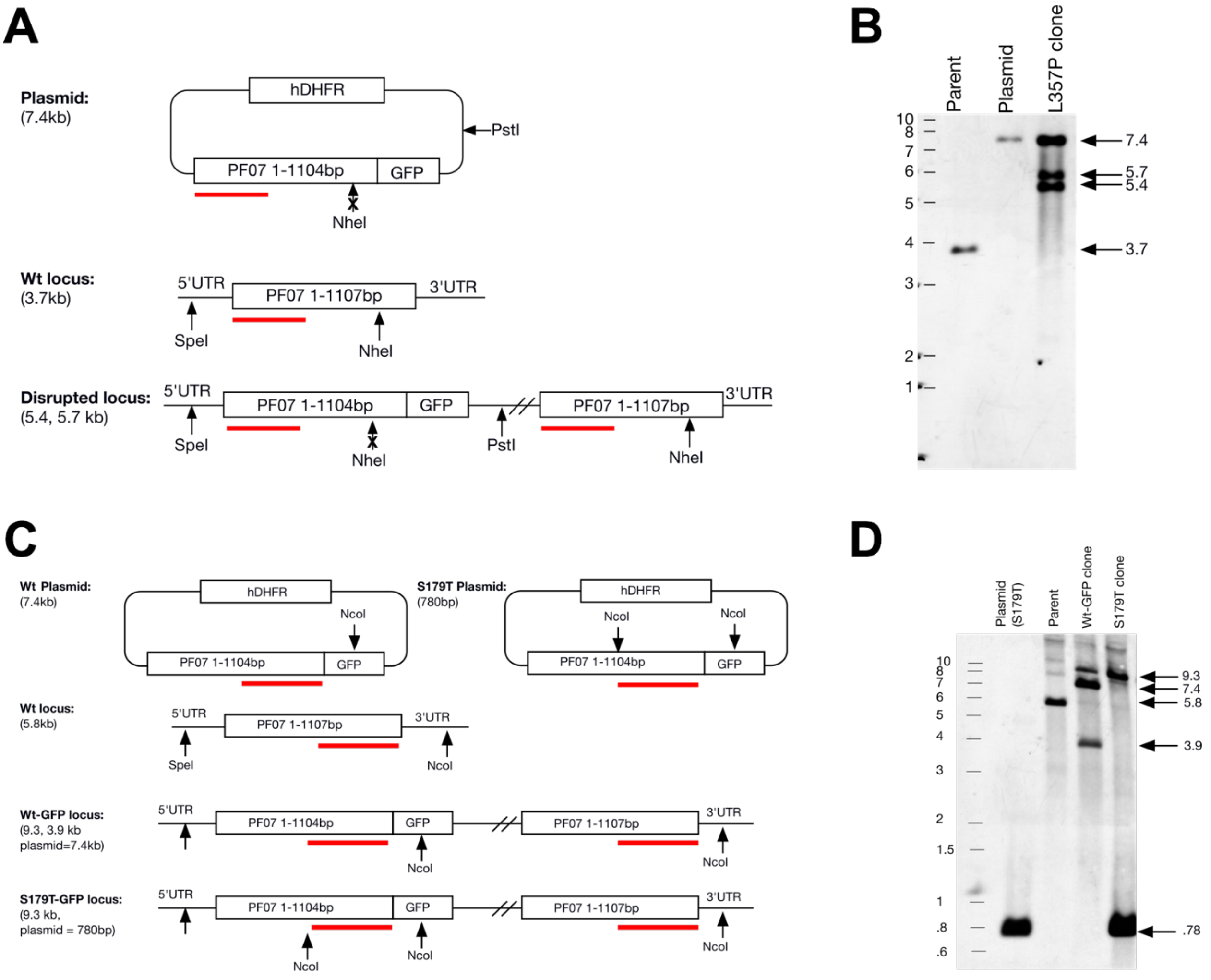

Supplementary Fig. 1: Introduction of Resistance-Conferring Mutations in PF3D7_0709700

A. Schematic of the SpeI, NheI, PstI digest used to diagnose the generation of L357P-GFP parasites. The mutation removes the NheI site present in the wild-type locus. The location of the PCR product used as a probe is in red.

B. Southern blot of gDNA from clonal parasites digested with SpeI, NheI, PstI. DNA ladder marked in kb.

C. Schematic of the SpeI, NcoI digest used to diagnose the generation of Wt-GFP and S179T-GFP clones. The wild-type plasmid has a single NcoI site, while the mutant plasmid contains 2 NcoI sites (one immediately 5' of the mutation). The location of the PCR product used as a probe is in red.

D. Southern blot of gDNA from clonal parasites digested with SpeI and NcoI.

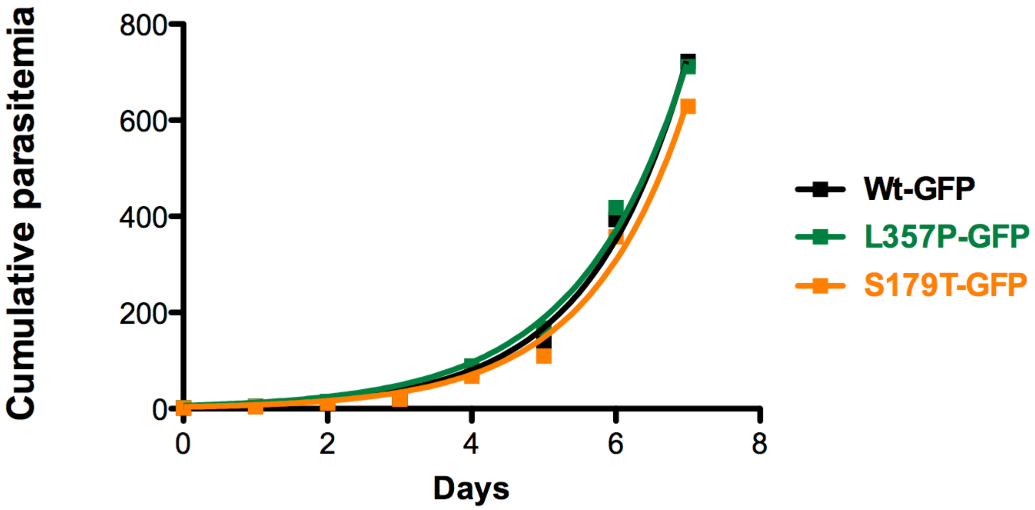

Supplementary Fig. 2: PfPARE is Not Essential for Asexual Parasites
Growth of GFP-tagged wild-type or mutant *pfpare*-expressing parasites was followed for one week. Parasitemia (percentage of total erythrocytes infected with parasites) as a function of time was fit to an exponential growth equation. 95% confidence intervals of doubling times (in days) are as follows: Wt-GFP=0.88 to 1.01, L357P-GFP=0.96 to 1.11, S179T-GFP=0.87 to 1.04. Wt and mutant growth constants are not different (F-test; $p = 0.14$). Error bars are s.d for triplicates of the representative experiment shown.N=2.

a

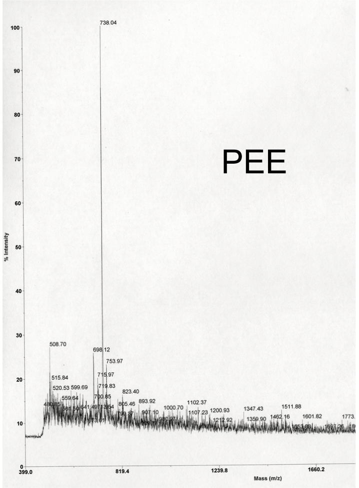

b

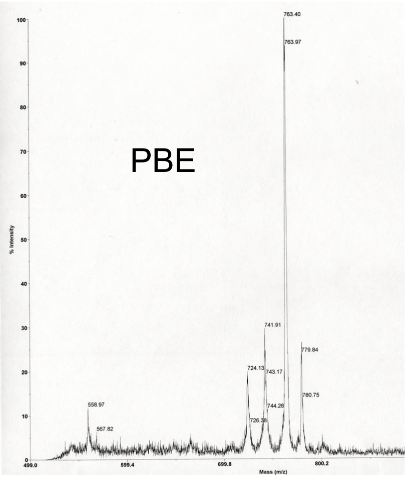

c

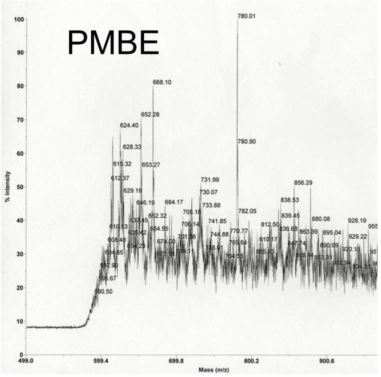

d

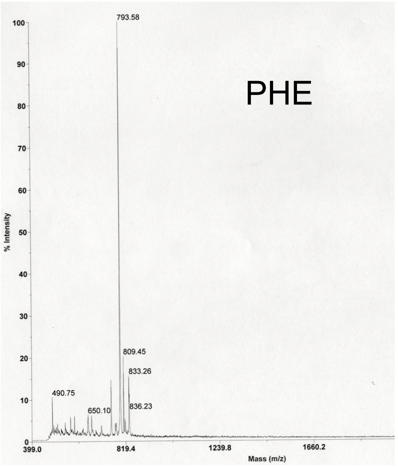

e

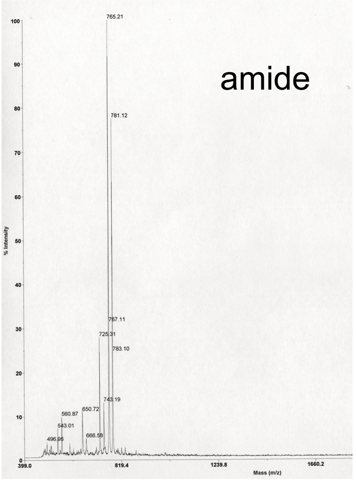

Supplementary Fig. 3: MALDI-TOF Spectra of Synthesized Compounds
a. PEE, b. PBE, c. PMBE, d. PHE, e. pepstatin n-butyl amide

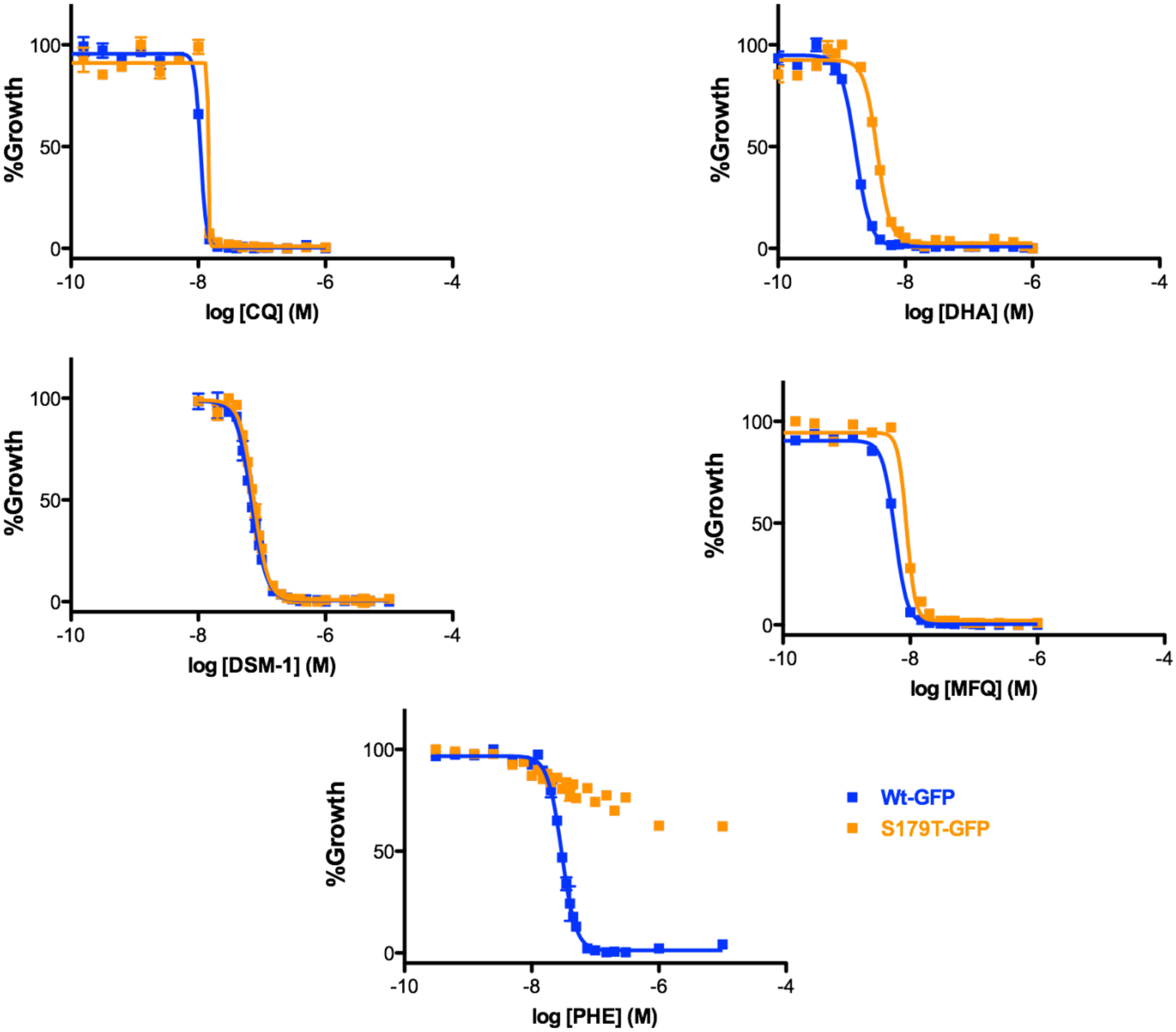

Supplementary Fig. 4: Concentration-response curves of Wt-GFP and S179T-GFP expressing parasite clones with well-characterized antimalarials
CQ (chloroquine), DHA (dihydroartemisinin), DSM-1 (5-methyl[1,2,4]triazolo[1,5-a]pyrimidin-7-yl)naphthalen-2-ylamine), MFQ (mefloquine), PHE (pepstatin hexyl ester). Error bars are s.d for triplicates of the experiment shown. N=1.

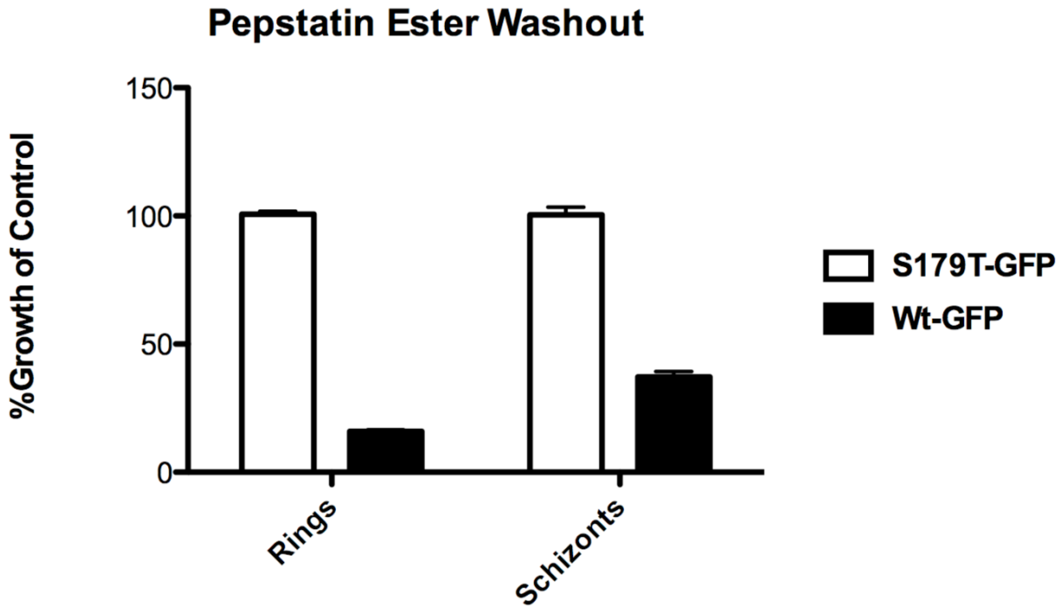

Supplementary Fig. 5: Pepstatin ester is more potent in young wild-type *pfpare*-expressing parasites

Synchronous ring or schizont-stage parasites were incubated with 2 μM PBE for 3 hours. PBE was removed by washout and ring parasitemia of the following cycle was measured using flow cytometry. Data were normalized to growth in vehicle (DMSO). S179T-expressing parasites are resistant to PBE (clear). Ring stage Wt-expressing parasites are more sensitive to PBE than schizont stage Wt-expressing parasites. Error bars are s.d.for triplicates of the experiment shown. N=1.

Supplementary Note 1

Listing of NMR peaks for n-hexyl ester of pepstatin

^{1}H NMR (400 MHz, CD_3OD): δ0.93-0.96 (m, 35H), 1.31-1.36 (m, 9H), 1.55-1.65 (m, 6H), 2.03-2.13 (m, 5H), 2.31-2.48 (m, 4H), 3.88-3.92 (m, 1H), 3.98-4.02 (m, 3H), 4.05 (t, *J*=6.6 Hz, 2H), 4.11 (d, *J*=7.8, 1H), 4.15 (d, *J*=8.2 Hz, 1H), 4.30 (q, *J*=7.1, 1H)

^{13}C NMR (CD_3OD) (100 MHz, CD_3OD): δ13.03, 16.97, 17.57, 17.76, 18.63, 18.69, 21.03, 21.09, 21.45, 22.28, 22.43, 24.51, 24.56, 25.40, 26.15, 28.36, 30.10, 30.23, 31.31, 38.76, 39.90, 40.03, 44.65, 49.53, 51.04, 51.36, 59.32, 59.59, 64.38, 69.58, 69.68, 172.15, 172.45, 172.76, 174.01, 174.41.

Supplementary Note 2: Sequence of codon-optimized PF3D7_0709700

ATGCTCGAGGACGGTAATCCAAAACTGGATAGCTTTCATAACAAAGATGGTCTGTC
TCTGAAAACCTACGCGTGGACTGTGAAAAATCCGGTCGGTGTTATCATCGCATGTC
ACGGTATGAATTCCCACGTCCGTCTGGAGTACCTGCGCCACAACGTTGAAGTTGTC
AACAACAACAAGGCGATCCTGAAAGATGGCGACAACTACTATATCTACAAAAACTCT
TGGATCGAAGAGTTCAACAAAAACGGCTATTCCTTCTACGGCATCGACCTGCAATC
CCACGGCCAAAGCGAAGGTTGGAAAGGTCTGCGTACCCACATCCGTCAGTTCGAT
GATATTGTGTATGACTTCATTCAGTACATTAATCGTATCCATGACATGCTGTGCCTG
AAAAACAAAAAAGATAATAACTCTTCCCTGCACGATAACATCAACAACAACAACAAC
ATCCTGCCGTTTTACATTATGGGTCTGTCTATGGGTGGCAACGTTGTGCTGCGTAC
TCTGCAGATCCTGGGCAAATCCAAGGATAACAACAACAAACTGAATATCCGTGGTT
GTATCCCGCTGGCAGGTATGATCTCTATTGACGAACTGGCCACGAAACCATCTTAT
AAGTATTTCTATATCCCGCTGGCTAAGTTCCTGGGCTCCTTCTTCCCGAGCCTGCG
CCTGACCCCTGGCCTGCGCTTCAACATGTTCCCTCATATGAACGATATTATGGAGTT
CGATAAATTCAAGTTCAAGAAACACGTAACCTGCCGCCTGGGCTATGAACTGCTGA
ACGCGATCAACAACCTGAACAACGACATGGATTACATCCCGGAGAACACCCCGATC
CTGTTTGCGCATAGCAAAAAAGACTCTGTATGTTTCTATGGCGGTACCCTGAAATTC
TACAACAAGCTGAAATGCCTGAAAAAAGAACTGTACACTCTGGACGACATGGATCA
CCTGCTGCCGATGGAACCGGGTAATGAACGCGTGCTGAAAAAAATTATTACCTGGC
TGGCTGTTCACACTCCGAAGCAGGAAGAACAGGTATAACTCGAG
